# *De novo* Fatty Acid Biosynthesis Contributes Significantly to Establishment of a Bioenergetically Favorable Environment for Vaccinia Virus Infection

**DOI:** 10.1371/journal.ppat.1004021

**Published:** 2014-03-20

**Authors:** Matthew D. Greseth, Paula Traktman

**Affiliations:** Department of Microbiology & Molecular Genetics, Medical College of Wisconsin, Milwaukee, Wisconsin, United States of America; University of Pennsylvania, United States of America

## Abstract

The poxvirus life cycle, although physically autonomous from the host nucleus, is nevertheless dependent upon cellular functions. A requirement for *de novo* fatty acid biosynthesis was implied by our previous demonstration that cerulenin, a fatty acid synthase inhibitor, impaired vaccinia virus production. Here we show that additional inhibitors of this pathway, TOFA and C75, reduce viral yield significantly, with partial rescue provided by exogenous palmitate, the pathway's end-product. Palmitate's major role during infection is not for phospholipid synthesis or protein palmitoylation. Instead, the mitochondrial import and β-oxidation of palmitate are essential, as shown by the impact of etomoxir and trimetazidine, which target these two processes respectively. Moreover, the impact of these inhibitors is exacerbated in the absence of exogenous glucose, which is otherwise dispensable for infection. In contrast to glucose, glutamine is essential for productive viral infection, providing intermediates that sustain the TCA cycle (anaplerosis). Cumulatively, these data suggest that productive infection requires the mitochondrial β-oxidation of palmitate which drives the TCA cycle and energy production. Additionally, infection causes a significant rise in the cellular oxygen consumption rate (ATP synthesis) that is ablated by etomoxir. The biochemical progression of the vaccinia life cycle is not impaired in the presence of TOFA, C75, or etomoxir, although the levels of viral DNA and proteins synthesized are somewhat diminished. However, by reversibly arresting infections at the onset of morphogenesis, and then monitoring virus production after release of the block, we determined that virion assembly is highly sensitive to TOFA and C75. Electron microscopic analysis of cells released into C75 revealed fragmented aggregates of viroplasm which failed to be enclosed by developing virion membranes. Taken together, these data indicate that vaccinia infection, and in particular virion assembly, relies on the synthesis and mitochondrial import of fatty acids, where their β-oxidation drives robust ATP production.

## Author Summary

Vaccinia virus, the prototypic poxvirus, is closely related to variola virus, the etiological agent of smallpox. A full understanding of the poxviral life cycle is imperative for the development of novel antiviral therapies, the design of new vaccines, and the effective and safe use of these viruses as oncolytic agents. Metabolomic studies have shed light on the novel mechanisms used by viruses to replicate efficiently within their hosts. *de novo* fatty acid biosynthesis has been shown to be of relevance for numerous viral infections as well as for the development of cancer. Here we describe an important role for *de novo* fatty acid biosynthesis during vaccinia infection. Ongoing synthesis of palmitate is needed to fuel the production of energy within mitochondria. The biochemical events of viral DNA replication and protein synthesis are minimally affected by inhibition of this pathway, but viral assembly is disrupted more dramatically. Further exploration of this pathway will provide additional insight into the infectious cycle and inform new therapeutic strategies for this important class of pathogen.

## Introduction

Global cellular metabolism is an intricate and tightly regulated network of pathways that generate building blocks and energy to sustain the life of the cell. Viral infection often leads to shifts in substrate utilization, dysregulation of metabolic pathways and changes in cellular energetics to facilitate maximal viral replication. This is exemplified by studies conducted on human cytomegalovirus (HCMV), in which infected cells display augmented cellular metabolism [1], [2] and an anaplerotic shift in which glucose is converted into precursors for nucleotide and lipid synthesis and glutamine is utilized to replenish the tricarboxylic acid (TCA) cycle [3].

**Figure 1:**
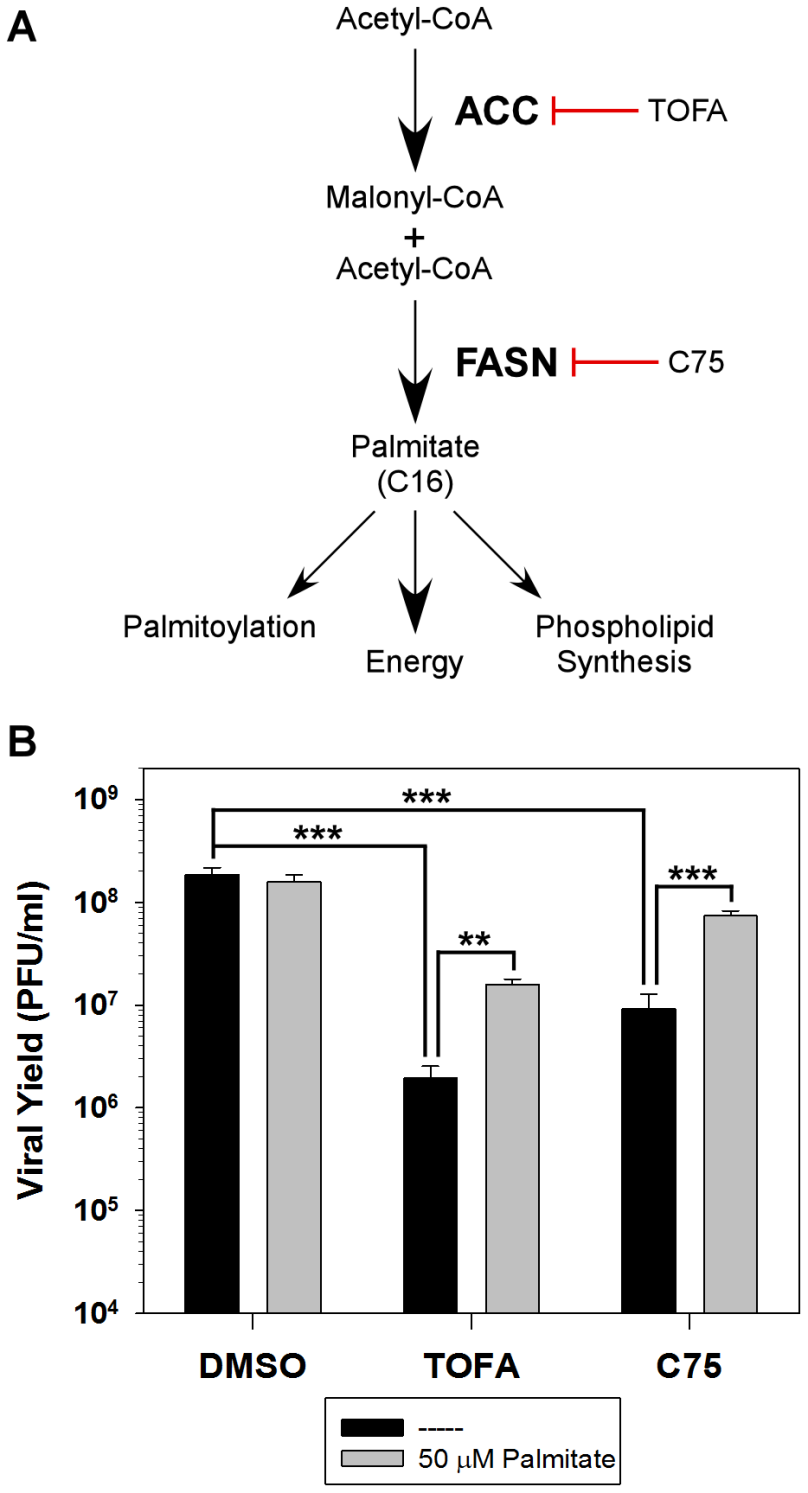
Analysis of the *de novo* fatty acid biosynthesis pathway during vaccinia infection. A) Schematic representation of the *de novo* fatty acid biosynthetic pathway and the biological functions of palmitate. Key enzymes in the pathway are highlighted in bold [acetyl-CoA carboxylase (ACC) and fatty acid synthase (FASN)] as well as their cognate inhibitors TOFA and C75, respectively. B) One-step growth analysis of BSC40 cells infected with WT vaccinia virus (MOI 5) in the presence of DMSO, TOFA (154 µM) or C75 (39 µM) in the absence (black bars) or presence (grey bars) of supplemental, exogenous palmitate (50 µM); viral yield at 16 hpi is shown (n = 6) (**, p<0.02; ***, p<0.001).

In addition to virally induced changes in overall metabolism, specific intermediate pathways can be exploited during infection. *De novo* fatty acid biosynthesis has been shown to be manipulated by several diverse viruses such as HCMV, dengue virus (DV) and Hepatitis C virus (HCV). The *de novo* fatty acid biosynthesis pathway generates the long-chain fatty acid palmitate and is schematically represented in Figure 1A. The first committed step in this pathway is achieved by the conversion of acetyl-CoA to malonyl-CoA by acetyl-CoA carboxylase (ACC). Subsequently, successive condensation reactions of malonyl-CoA with acetyl-CoA are catalyzed by fatty acid synthase (FASN), ultimately generating the 16-carbon fatty acid palmitate. Palmitate contributes to several key biological functions such as protein palmitoylation, phospholipid synthesis and energy production (**Fig. 1A**) [4].

HCMV increases the activity of ACC as a means of upregulating phospholipid synthesis, thereby providing additional membranes for viral assembly [5]. Similarly, the DV protein NS3 has been shown to actively relocalize FASN to sites of viral replication and increase rates of fatty acid synthesis [6]. HCV has been shown to upregulate expression of the two key enzymes in the *de novo* fatty acid biosynthesis pathway, ACC and FASN, in a sterol regulatory element binding protein-c (SREBP-c) dependent manner [7]–[10], as well as to relocalize FASN to sites of viral replication [11]. Both of these RNA viruses are known to replicate their genome on a scaffold of intracellular membranes. In the case of these three viruses, increased *de novo* fatty acid biosynthesis is utilized for increased phospholipid synthesis.

Vaccinia virus, the prototypic poxvirus, was used as the vaccine for the successful eradication of smallpox. The large size of the viral genome, which encodes approximately 200 genes, enables the virus to replicate solely in the cytoplasm of infected cells. The array of virally encoded proteins include those that mediate viral entry, three temporally regulated phases of gene expression, genome replication and maturation, virion morphogenesis and egress, and a range of interactions with the intrinsic, innate and adaptive defenses of the host. Despite the virus' physical and genetic autonomy from the host nucleus, infection still requires macromolecule precursors such as nucleotides, phospholipids, and amino acids as well as intracellular membranes and the host translational machinery. A comprehensive metabolomic analysis of vaccinia-infected cells is lacking; indeed very little is known about how vaccinia virus manipulates or interfaces with the host metabolome or bioenergetic machinery. Recently, the Smith lab reported that vaccinia induces a ‘pseudo hypoxic’ state early in infection [12]. We have also previously shown that the FASN inhibitor, cerulenin, diminishes viral yield [13], suggesting that *de novo* fatty acid biosynthesis is important for viral infection.

Here, we report our expanded analysis of the contribution of *de novo* fatty acid biosynthesis to vaccinia virus replication. Inhibition of this pathway impairs the production of infectious virus in a manner that can be partially rescued by the addition of exogenous palmitate. The mitochondrial import and β-oxidation of palmitate appear to be very important during infection, whereas exogenous glucose is dispensable, suggesting that the conversion of palmitate to acetyl-CoA, rather than the glycolytic processing of glucose, drives the TCA cycle and aerobic energy production. Additionally, glutamine is essential for vaccinia replication, serving to anaplerotically fill the TCA cycle. Examination of how the impairment of *de novo* fatty acid biosynthesis affected infection revealed the unimpeded progression through the biochemical stages of infection, a modest reduction in DNA and protein synthesis, and a more dramatic block to virion assembly. Cumulatively, our data are consistent with the conclusion that *de novo* fatty acid biosynthesis is essential for energy production, rather than phospholipid synthesis, during poxvirus infection.

## Results

### Palmitate is important for maximal viral replication

As mentioned above, several viruses manipulate the *de novo* fatty acid biosynthesis pathway to augment the synthesis of phospholipids and increase the availability of intracellular membranes needed for efficient replication. We have previously shown that the fatty acid synthase (FASN) inhibitor, cerulenin, inhibits viral yield [13], suggesting that this pathway may also be important for vaccinia virus infection. To further assess the contribution of this pathway to vaccinia infection, the impact of two additional pharmacological inhibitors was assessed: 5-(Tetradecyloxy)-2-furoic acid (TOFA) and C75, which inhibit the cellular enzymes ACC and FASN, respectively (**Fig. 1A**). BSC40 cells were infected with WT vaccinia virus and treated with vehicle control (DMSO), TOFA or C75 for 16 h at 37°C. Treatment with TOFA or C75 significantly inhibited viral yield by 95- and 20-fold, respectively (**Fig. 1B**, black bars).

To validate the specificity of these inhibitors, we tested the ability of the end-product of the *de novo* fatty acid biosynthetic pathway, palmitate, to reverse their impact. Palmitic acid was coupled to fatty-acid free bovine serum albumin [14] and 50 µM exogenous palmitate was added to infected cells treated with DMSO, TOFA, and C75 as described above. Addition of palmitate had no impact on cells treated with DMSO, but rescued viral yield by 8-fold in cells treated with either TOFA or C75 (**Fig. 1B**, gray bars).

Immunoblot analysis of lysates prepared from cells infected under these conditions showed that the accumulation of the viral early protein I3 was unaffected by the inhibitors tested. However, there was a decrease in the accumulation of the viral late protein F18 and a decrease in the cleavage of the viral protein L4 (a hallmark of virion assembly); both of these inhibitors' effects were ameliorated by the addition of exogenous palmitate (data not shown). Taken together, these data show that the *de novo* fatty acid biosynthetic pathway, and palmitate specifically, are important for maximum viral production.

### The key role for palmitate during viral replication is not for post-translational modification of proteins or as a substrate for phospholipid synthesis

**Figure 2:**
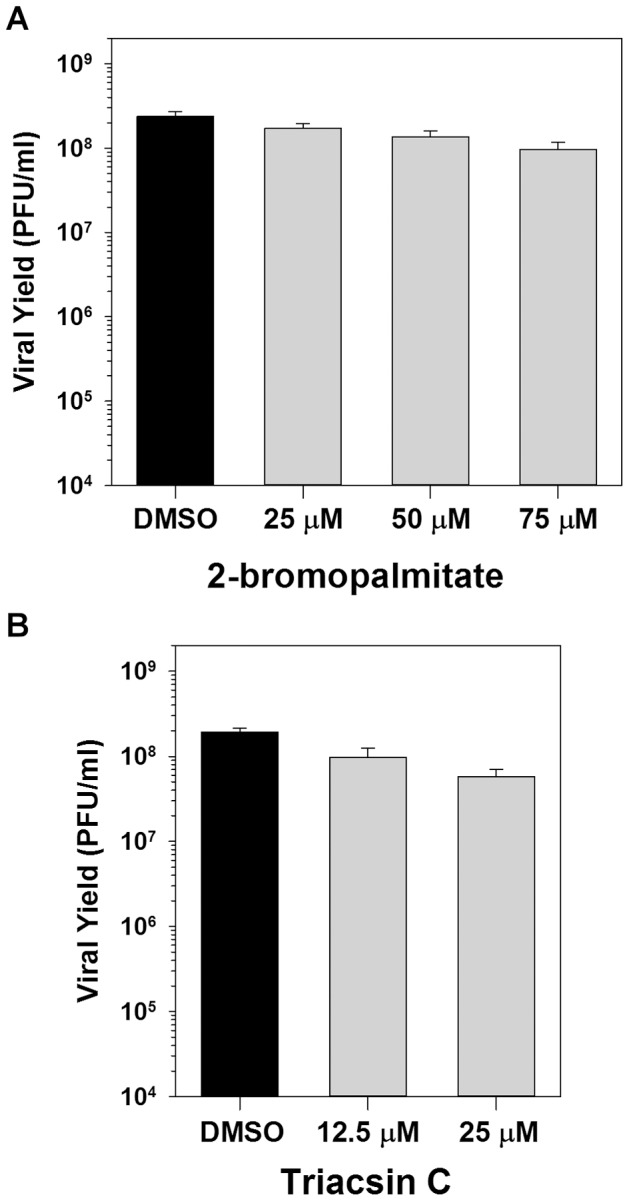
Inhibition of palmitoylation or phospholipid synthesis has no impact on viral replication. A) One-step growth analysis of BSC40 cells infected with WT vaccinia virus (MOI 5) in the presence of various concentrations (0, 25, 50 and 75 µM) of 2-bromopalmitate, a palmitate analogue and inhibitor of palmitoylation; viral yield at 16 hpi is shown (n = 6). B) One-step growth analysis of BSC40 cells infected with WT vaccinia virus (MOI 5) in the presence of various concentrations (0, 12.5 and 25 µM) of triacsin C, an inhibitor of fatty acid chain elongation and hence phospholipid synthesis; viral yield at 16 hpi is shown (n = 6).

Palmitate has several biological functions within the cell as shown in Figure 1A: protein palmitoylation, phospholipid synthesis or energy production via ß-oxidation. To determine if palmitate is utilized for palmitoylation, BSC40 cells were infected in the presence of vehicle (DMSO) or various concentrations of the palmitate analogue 2-bromopalmitate, which impairs palmitoylation by inhibiting palmitoyl acyl transferases [15]. Inclusion of 25–75 µM 2-bromopalmitate had no impact on the 16 h viral yield (**Fig. 2A**), whereas coronavirus replication is inhibited by ≤10 µM [16]. To validate the efficacy of this inhibitor in our hands, BSC40 cells were incubated with [^3^H]-palmitate for 4 h in the presence of DMSO or 50 µM 2-bromopalmitate, and whole cell lysates were resolved and analyzed by fluorography [17]. As expected, the levels of [^3^H]-palmitoylated proteins was greatly decreased in the 2-bromopalmitate-treated sample (**Fig S1A**).

We next determined whether the utilization of palmitate for phospholipid synthesis is important for viral replication by assessing the impact of the pharmacological inhibitor triacsin C. Triacsin C is a potent competitive inhibitor of long chain acyl-CoA synthetase, and thus prevents the acylation of long chain fatty acids and hence the initiation of phospholipid synthesis [18]. Treatment with 12.5 or 25 µM triacsin C had no impact on the 16 h viral yield (**Fig. 2B**). To validate the efficacy of this inhibitor, BSC40 cells were incubated with 400 µM oleic acid in the presence of DMSO or 6.25 µM triacsin C, and stained with BODIPY493/503 to monitor the inclusion of oleic acid into lipid droplets, a process that requires acylation of oleic acid by acyl-CoA synthetase [19]–[22]. Treatment with 6.25 µM triacsin C abrogated the formation of lipid droplets (**Fig S1B**). Of note, concentrations between 1 and 10 µM triacsin C have previously been shown to inhibit rotavirus replication [19], [23]. Taken together, these data indicate that the importance of palmitate synthesis (and fatty acid biosynthesis in general) during vaccinia infection is independent of its contributions to protein modification and phospholipid synthesis.

### Mitochondrial import and ß-oxidation of palmitate are important for viral infection; glucose, however, is dispensable

**Figure 3:**
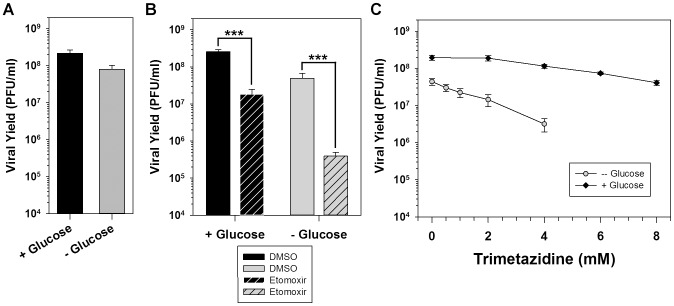
Palmitate is utilized within mitochondria for energy production via ß-oxidation. A) One step growth analysis of BSC40 cells infected with WT vaccinia virus (MOI 5) in glucose-free media (gray bar) or glucose-free media supplemented with 4 mM glucose (black bar); viral yield at 16 hpi is shown (n = 6). B) One step growth analysis of BSC40 cells infected with WT vaccinia virus (MOI 5) in the presence (left bars) or absence (right bars) of 4 mM glucose supplemented with DMSO (vehicle control) (solid bars) or etomoxir (360 µM) (striped bars), an inhibitor of long-chain fatty acid import into the mitochondria; viral yield at 16 hpi is shown (n = 6) (***, p<0.001). C) One-step growth analysis of BSC40 cells infected with WT vaccinia virus (MOI 5) in the presence (black diamonds) or absence (gray circles) of 4 mM glucose and treated with various concentrations (0–8 mM) of trimetazidine, an inhibitor of ß-oxidation; viral yield at 16 hpi is shown (n = 6).

The data presented above suggest that the key contribution of palmitate during vaccinia infection might be its potential to undergo β-oxidation and promote energy production. As a first approach to understanding the bioenergetics pathways that contribute to productive viral infection, we tested the importance of glucose, which can be metabolized to pyruvate and ultimately acetyl-CoA, thereby driving the TCA cycle. BSC40 cells were therefore infected with WT vaccinia virus in glucose-free media or media supplemented with 4 mM glucose (**Fig. 3A**, gray and black, respectively). Surprisingly, the absence of glucose had no impact on the 16 h viral yield, suggesting that infected cells might be relying on ß-oxidation to drive the TCA cycle and oxidative phosphorylation, rather than glycolysis, to provide energy.

To directly test the hypothesis that infected cells rely on the import and utilization of palmitate within mitochondria, cells were infected in the presence of vehicle (solid bars) or the pharmacological inhibitor etomoxir (striped bars). Etomoxir is an irreversible inhibitor of carnitine palmitoyltransferase I (CPT1) and prevents the import of long-chain fatty acids into mitochondria. Treatment with etomoxir inhibited the 16 h viral yield 14-fold compared to treatment with DMSO (**Fig. 3B**, left bars). As expected based on the mode of action of etomoxir, the addition of 50 µM exogenous palmitate was unable to rescue viral yield (data not shown). Intimate feedback between the various facets of cellular metabolism is illustrated by the observation that inhibition of β-oxidation causes a concomitant increase in the glycolytic pathway [24]. In light of this interrelatedness, the impact of etomoxir treatment on viral replication in cells maintained in the absence of glucose was also examined. When glucose was omitted from the medium, treatment with etomoxir caused a much greater decrease (125-fold) in viral yield than had been seen in glucose-containing media (**Fig. 3B**, right bars). These data show that import of long-chain fatty acids into mitochondria is important for viral infection.

To formally test the hypothesis that virus infection requires ß-oxidation of long-chain fatty acids within mitochondria for energy production, cells were infected in the presence of various concentrations of the pharmacological inhibitor trimetazidine (TMZ). TMZ is a competitive inhibitor of 3-ketoacyl coenzyme A thiolase, a key enzyme in ß-oxidation [25]. Inclusion of 2–8 mM TMZ led to a dose-dependent, but modest, decrease in the 16 h viral yield when cells were maintained in the presence of glucose [5-fold decrease at 8 mM (**Fig. 3C**, black diamonds)]. As was seen with etomoxir (**Fig. 3B**), treatment with TMZ in the absence of glucose caused a dose-dependent and more severe inhibition in viral yield [36-fold decrease at 4 mM (**Fig. 3C**, gray circles)]. Taken together, these data show that the import of palmitate into mitochondria and its subsequent β-oxidation, are important for viral infection. In addition, the presence of glucose is not important for viral replication unless β-oxidation of fatty acids is inhibited, suggesting that the TCA cycle within infected cells is normally driven by acetyl-CoA generated by the ß-oxidation of palmitate, rather than from glycolysis.

### Glutamine plays a critical role during infection that can be partially substituted by addition of TCA cycle intermediates

**Figure 4:**
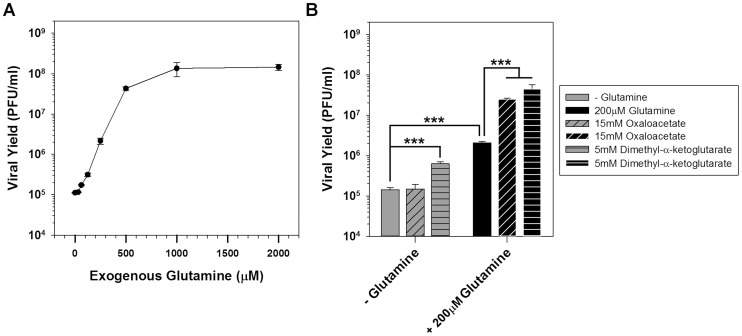
Glutamine is essential for vaccinia infection and anaplerotically supplements the TCA cycle. A) One step growth analysis of BSC40 cells infected with WT vaccinia virus (MOI 5) in glutamine-free media supplemented with various concentrations of glutamine; viral yield at 16 hpi is shown (n = 2). B) Grey bars: one step growth analysis of BSC40 cells infected with WT vaccinia virus (MOI 5) in glutamine-free media or glutamine-free media supplemented with 15 mM oxaloacetate (diagonal stripes) or 5 mM dimethyl-α-ketoglutaric acid (horizontal stripes). Black bars: one step growth analysis of BSC40 cells infected with WT vaccinia virus (MOI 5) in medium containing 200 µM glutamine, or media containing 200 µM glutamine supplemented with 15 mM oxaloacetate (diagonal stripes) or 5 mM dimethyl-α-ketoglutaric acid (horizontal stripes). Viral yield at 16 hpi is shown (n = 4); *** denotes p<0.001).

As an alternative to glycolysis, the process of glutaminolysis can provide TCA cycle intermediates through the deamination of glutamine to glutamate, which is imported into mitochondria and converted into α-ketoglutarate. To evaluate the importance of glutamine for viral infection, BSC40 cells were infected with WT vaccinia virus in glutamine-free media or media supplemented with various concentrations of glutamine from 31.2–2000 µM (**Fig. 4A**). In the absence of glutamine, viral yield was reduced ∼1300-fold. The addition of 250 µM or 500 µM glutamine rescued viral yield by 20-fold and 384-fold, respectively; maximum virus production was restored at ≥1 mM glutamine.

To determine whether the TCA cycle was being sustained by the anaplerotic utilization of glutamine, cells were infected with WT vaccinia virus in glutamine-free medium or media supplemented with the TCA cycle intermediates oxaloacetate (diagonally striped bars) or α-ketoglutarate (horizontally striped bars) (**Fig. 4B**). In the absence of glutamine, the addition of 15 mM oxaloacetate was unable to rescue viral yield, and 5 mM dimethyl-α-ketoglutarate only rescued viral yield 4-fold. In the presence of 200 µM glutamine (which itself rescued viral yield 14-fold), the addition of 15 mM oxaloacetate or 5 mM dimethyl-α-ketoglutarate led to additional increases in virus production of 12- and 22-fold, respectively. Taken together, these data demonstrate that glutamine plays critical roles during infection, among which is the anaplerotic support of the TCA cycle, which can be sustained by an exogenous supply of oxaloacetate or α-ketoglutarate.

### Vaccinia virus infection leads to elevated ATP synthesis that is ablated by the addition of etomoxir

**Figure 5:**
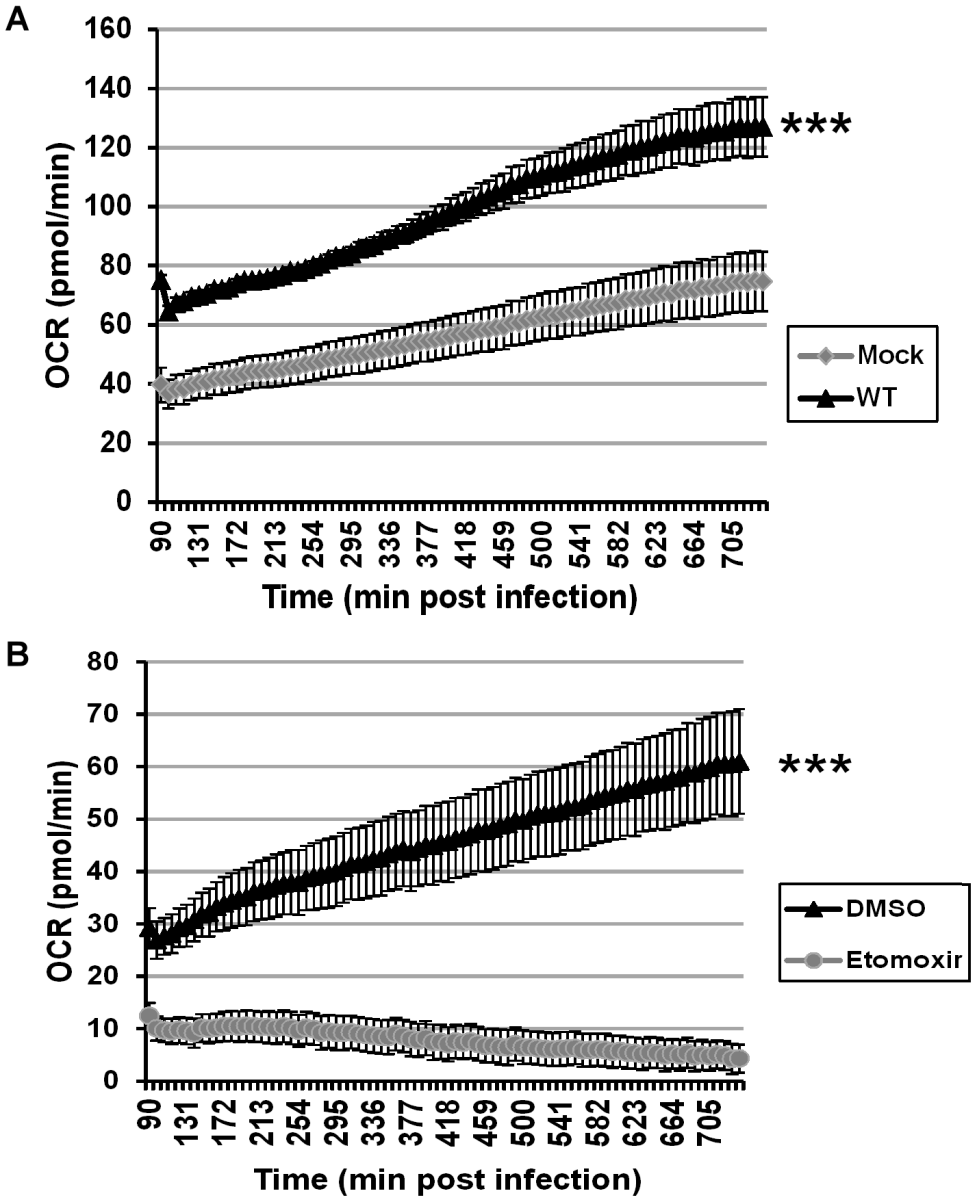
Vaccinia infection is accompanied by an increase in ATP synthesis that is ablated by etomoxir. Seahorse technology was employed for real-time, continual measurement of oxygen consumption rates (OCR) in cultures of uninfected or infected cells as a surrogate assessment of ATP synthesis. A) BSC40 cells were either mock infected (gray diamonds) or infected with WT vaccinia virus (MOI 5) (black triangles), and OCR was measured from 1.5 to 12 hpi (n = 12) (***, p<0.001). B) BSC40 cells were infected with WT vaccinia virus (MOI 5) in the presence of vehicle control (DMSO) (black triangle) or etomoxir (360 µM) (gray circles) and OCR was measured from 1.5 to 12 hpi (n = 12) (***, p<0.001).

Since it appears that the key role for palmitate during viral infection is to drive the TCA cycle and energy production, ATP synthesis was monitored during the course of infection. We utilized a Seahorse Bioscience XF-96 extracellular flux analyzer, which measures oxygen consumption rates as a direct surrogate of ATP synthesis [26]. This assay allows for repeated monitoring of ATP synthesis of a single sample over time. BSC40 cells were plated at a density of 40,000 cells per well (96-well dish) and were either mock infected or infected with WT vaccinia virus. Oxygen consumption rates were measured every 8 min from 90 min to 12 h post-infection (hpi). At 90 min post infection, mock treated cells had an oxygen consumption rate (OCR) of 40 pmol/min, which rose to 75 pmol/min by 12 hpi as nutrients were consumed (**Fig. 5A**, gray line). Strikingly, the initial OCR (90 min post-infection) of WT infected cells was almost twice that of mock infected cells at 75 pmol/min; this rate remained high throughout the infection, rising to 127 pmol/min at 12 hpi (**Fig. 5A**, black line). Statistical analysis indicated that the OCR was significantly higher in infected cells than mock-infected cells at all time points, and linear regression analysis indicated that the rate of increase in the OCR over the 10.5 hours of measurement was also significantly higher for the infected cells than that measured for mock-infected cells. Because the extracellular acidification rate, a readout of lactate production, remains largely unchanged over time (data not shown), this increase in OCR can be ascribed to mitochondrial rather than glycolytic activity.

To confirm that the elevated OCR seen in infected cells was due to increased ß-oxidation of palmitate and TCA cycle-driven ATP production, cells were infected with WT virus in the presence of vehicle or etomoxir [27] and subjected to Seahorse analysis. In DMSO-treated cells, the OCR time course had a similar slope as seen above (**Fig. 5B**, black line), while the OCR measurements in etomoxir-treated cells were depressed at all times and decreased over the course of infection (**Fig. 5B**, gray line). Taken together, these data show that even by 90 min post infection, oxygen consumption rates, and hence ATP synthesis, have increased almost 2-fold over mock treated cells and that this increase is maintained throughout infection. The loss of oxygen consumption seen in the presence of etomoxir confirms that the mitochondrial import of long-chain fatty acids is key for sustained and elevated ATP production within infected cells.

### A portion of FASN localizes to mitochondria and remains associated upon infection

**Figure 6:**
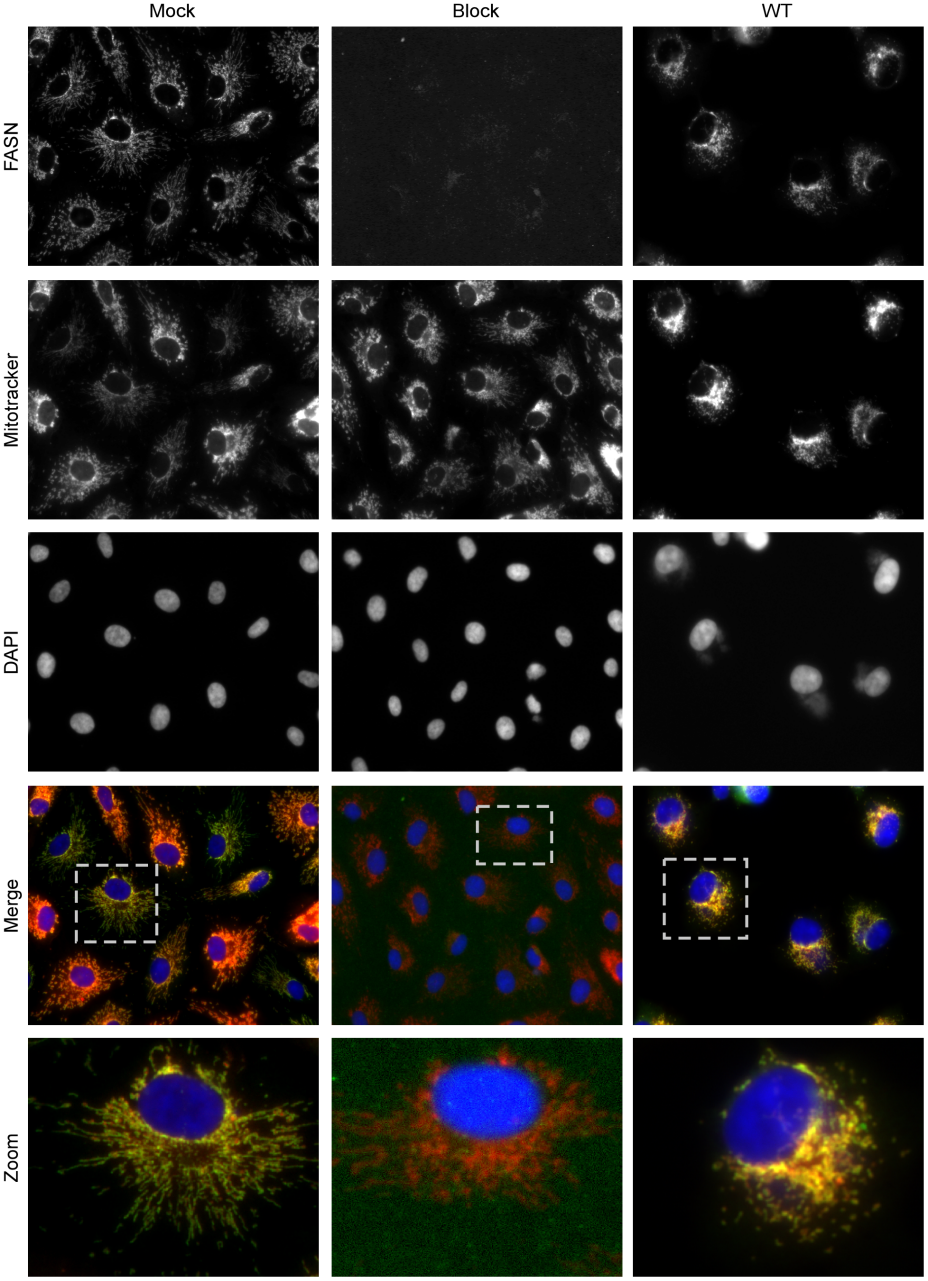
Immunofluorescence analysis of FASN localization in mock and virally infected cells. BSC40 cells were either mock infected (left and middle columns) or infected with WT vaccinia virus (MOI 2) (right column) for seven hours and fixed with cold methanol∶acetone (1∶1). Samples were stained with primary antiserum recognizing FASN and blocking peptide (as appropriate) followed by AlexaFluor488 secondary antibody (top row) as well as the cell permeable dyes Mitotracker Red (to stain the mitochondria) (second row) and DAPI (to stain DNA) (third row). Merged images of FASN, Mitotracker and DAPI are shown in the fourth row. A single cell is shown at higher magnification in the bottom row and corresponds to the cell outlined in the dotted box (fourth row). Merged images show the ablation of FASN staining when a blocking peptide was included, and the significant colocalization of FASN with the mitochondria, both in mock and infected samples.

Previously, FASN has been reported to have a diffuse localization within cells as assessed by immunofluorescence microscopy [11], and FASN has been shown to relocalize to sites of viral replication in cells infected DV or HCV [6], [11]. We utilized a variety of commercially available antibodies to monitor the localization of FASN in uninfected and vaccinia-infected BSC40 cells (7 hpi) by immunofluorescence microscopy. The most reproducible and distinct pattern was seen using a polyclonal antibody raised against a peptide from the C′-terminus of FASN. In mock-infected cells (**Fig. 6**, left column), a tubular pattern of staining that radiated throughout the cytoplasm was seen. This pattern was reminiscent of mitochondrial staining, and indeed the anti-FASN staining pattern largely colocalized with the Mitotracker signal [merge (fourth row) and zoom (bottom row)] (Pearson's coefficient of 0.80). Validation of the specificity of the staining seen with the anti-FASN antibody was obtained by blocking cells with the peptide used as the immunogen; in the presence of this blocking peptide, no anti-FASN staining was seen (**Fig. 6**, middle column), although the Mitotracker (mitochondria) and DAPI (nuclei) staining remained unchanged. At 7 hpi with vaccinia virus, the pattern of anti-FASN staining was quite similar and remained coincident with the Mitotracker profile (Pearson's coefficient of 0.95), although both FASN and mitochondria were more concentrated in the perinuclear region of the cell (**Fig. 6**, right column). These data show that at least a portion of FASN, that portion most accessible to this anti-FASN antibody, localizes at or near the mitochondria, suggesting that palmitate may be generated by FASN in close proximity to mitochondria, facilitating its import by CPT1.

### TOFA, C75, and etomoxir have the greatest impact on the post-replicative phase of the vaccinia life cycle

**Figure 7:**
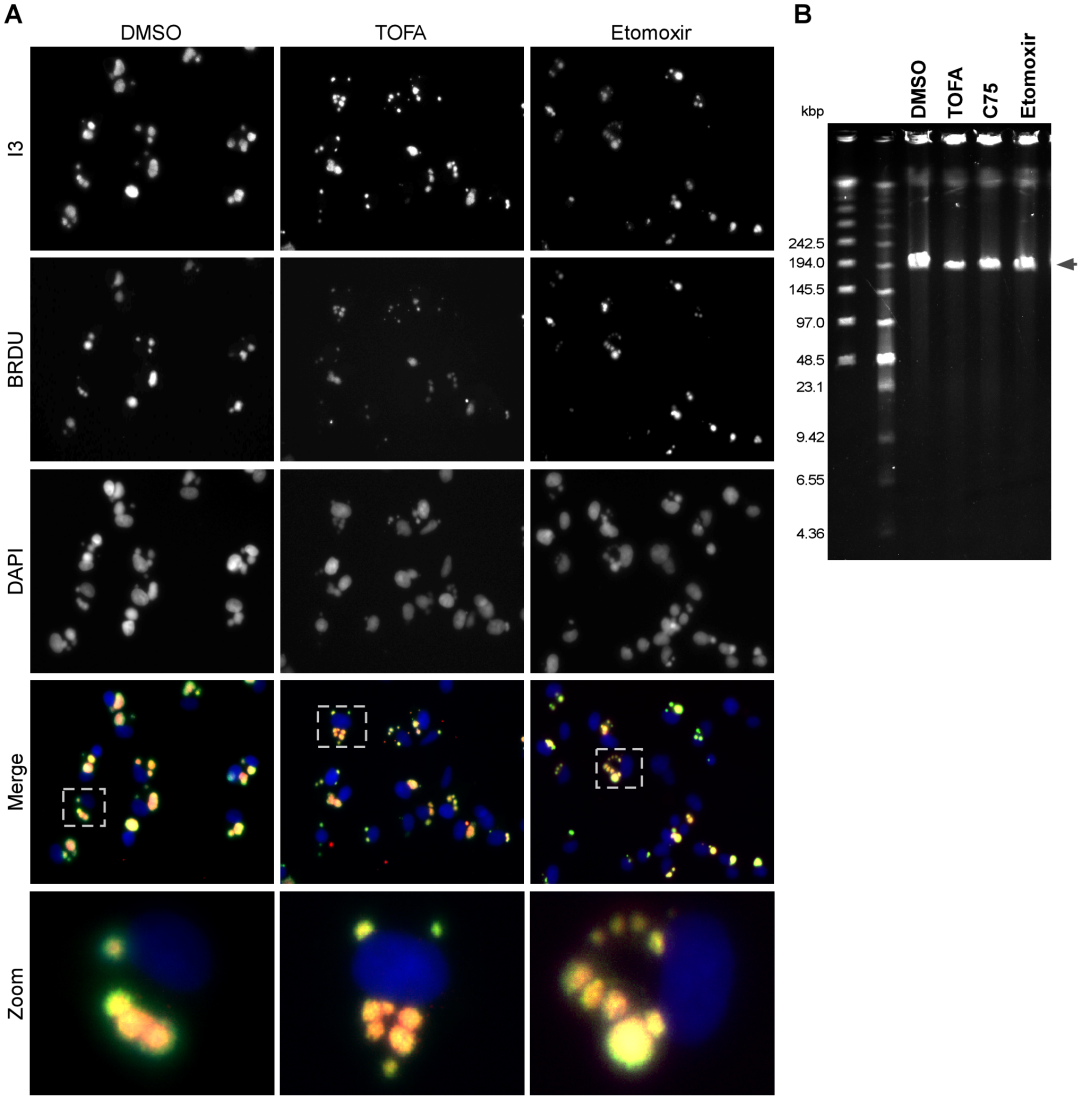
Viral DNA replication is mildly impacted by TOFA, C75 or etomoxir. A) BSC40 cells were infected with WT vaccinia virus (MOI 2) in the presence of 25 µg/ml BrdU, to track nascent DNA synthesis, as well as DMSO (left column), TOFA (154 µM) (middle column) or etomoxir (360 µM) (right column). At 7 hpi, cells were fixed and stained with primary antisera recognizing the viral I3 protein (top row), a marker of DNA replication sites, and BrdU (second row) as well as DAPI (third row). Merged images of I3, BrdU and DAPI are shown in the fourth row. A single cell is shown at higher magnification in the bottom row and corresponds to the cell shown in the dotted box (fourth row). Merged images show accumulation of replication foci under all treatments, although the foci seen in TOFA or etomoxir treated cells appear smaller and more numerous. B) Pulsed-field gel electrophoresis was employed to examine the accumulation and integrity of full length viral genomes. BSC40 cells were infected with WT vaccinia virus in presence of DMSO, TOFA (154 µM), C75 (39 µM) or etomoxir (360 µM). At 10 hpi, cells were processed for PFGE; DNA was visualized by ethidium bromide staining. Under all conditions tested, full length genomes accumulated; however in TOFA-, C75- and etomoxir-treated cells the levels of full-length genomic DNA that accumulated were 50, 70 and 83% (respectively) of that seen when cells were treated with vehicle (DMSO) alone.

Cumulatively, the data shown above indicate that the production, mitochondrial import, and β-oxidation of palmitate are important for productive vaccinia infection. As a first step in determining which stages of the viral life cycle were most affected by these inhibitors, we analyzed viral DNA replication on a per-cell basis (**Fig. 7A**). BSC40 cells were infected with WT vaccinia virus in the presence of BrdU (which is incorporated into newly synthesized DNA) and treated with DMSO, TOFA, or etomoxir for 7 h. Cells were then stained with antisera against BrdU [to mark sites of nascent viral DNA synthesis (**Fig. 7A**, second row)], I3 [the viral single-stranded DNA binding protein (**Fig. 7A**, top row)] and DAPI [to stain all DNA (**Fig. 7A**, middle row)]. In cells treated with vehicle (DMSO, left column), large sites of viral DNA replication accumulate in the cytoplasm as shown by the colocalization of I3 and BrdU [merge (fourth row) and zoom (bottom row)]. Similar replication foci were observed in cells treated with either TOFA (middle column) or etomoxir (right column), indicating that genome replication was proceeding in the presence of these inhibitors. Of note, the replication foci in the drug-treated cells appeared to be somewhat smaller and more numerous.

To examine the integrity of the viral DNA accumulated in the presence of the various drugs, pulsed-field gel electrophoresis was employed (**Fig. 7B**). BSC40 cells were infected with WT virus in the presence of DMSO, TOFA, C75 or etomoxir for 10 h. The DNA was resolved and visualized with ethidium bromide. Full-length viral genomes (∼200 kbp; arrowhead) were predominant under all treatments, although the levels of DNA accumulated were diminished to 50, 70 and 83% of WT levels in the presence of TOFA, C75 and etomoxir, respectively. Similar results were seen when viral DNA replication was assayed by Southern dot blot analysis (data not shown). Cumulatively, these data show that viral DNA replication is only modestly impaired when palmitate synthesis and mitochondrial import are impaired. Since DNA replication is accomplished by early viral proteins, and since the IF analysis (**Fig. 7A**) (and immunoblot assays, not shown) indicated that the I3 protein accumulates to high levels in the presence of these drugs, we can conclude that early protein synthesis is also largely unaffected by these inhibitors.

### Viral protein synthesis is modestly reduced in the presence of TOFA, C75, or etomoxir

**Figure 8:**
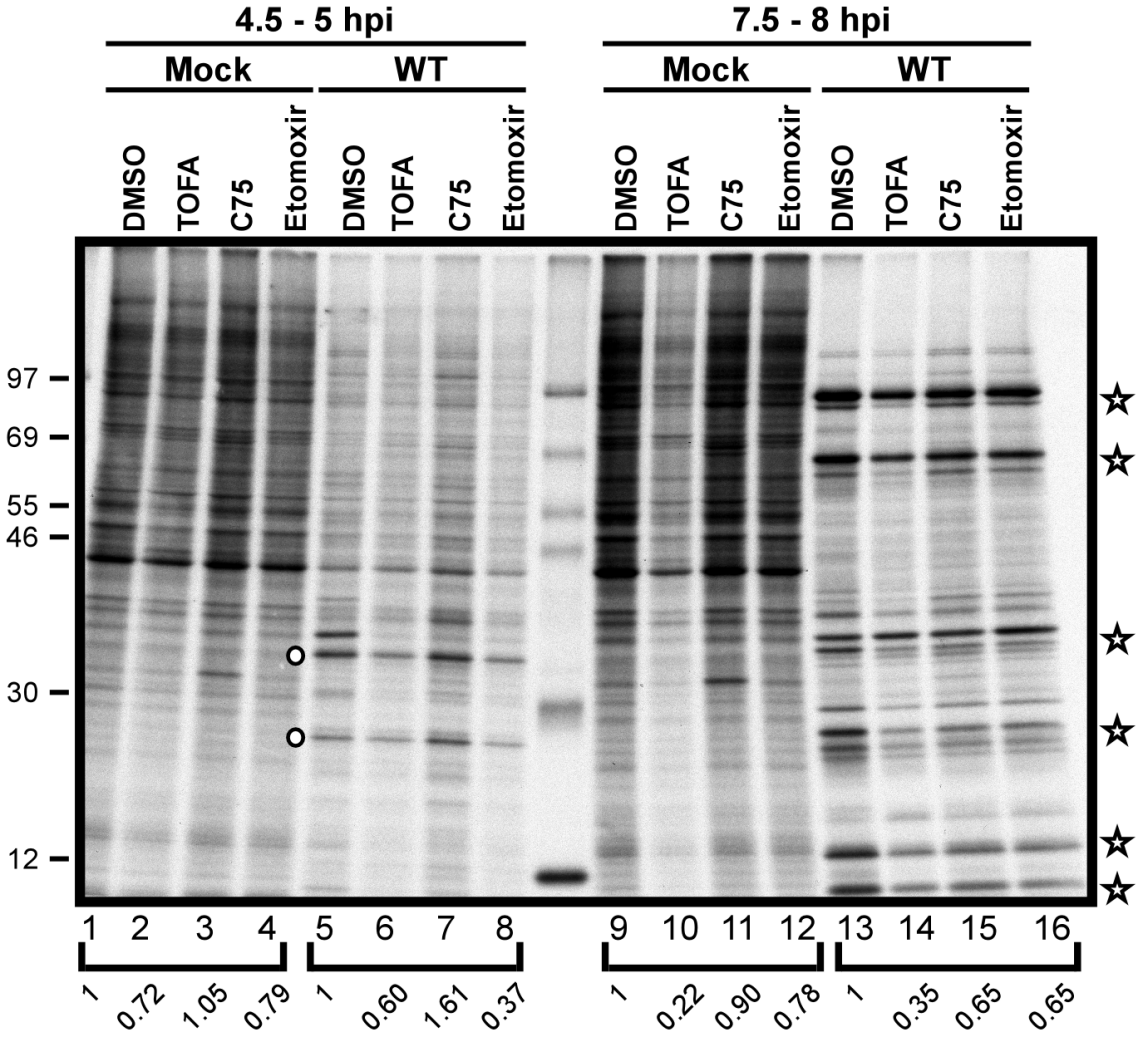
Viral protein synthesis is modestly reduced in the presence of TOFA, C75 and etomoxir. Protein synthesis was examined by pulsing both mock treated (lanes 1–4 and 9–12) and WT-infected (lanes 5–8 and 13–16) cells with 100 µCi/ml [^35^S]-Methionine for 30 min. Cells were maintained in the presence of vehicle (DMSO), TOFA (154 µM), C75 (39 µM) or etomoxir (360 µM) throughout the time course. At 4.5 hpi, WT infected cells showed a characteristic decrease in host protein synthesis as well as a concomitant appearance of intermediate protein synthesis (open circles, lanes 5–8). At later time points, WT infected cells show the synthesis of late viral proteins (open stars, lanes 13–16). Protein standards are shown at left with the molecular masses indicated in kDa. Quantification of protein synthesis was normalized to DMSO treated cells within each bracketed grouping.

Vaccinia virus infection is characterized by a temporally regulated cascade of gene expression, in which each phase of transcription (early, intermediate, late) is mediated by proteins expressed in the prior phase. Moreover, as infection progresses, host transcription is quelled, host mRNAs decrease in abundance, and translation is skewed towards viral mRNAs. We therefore assessed whether the progression and robustness of viral protein synthesis was impaired when the cycle of palmitate synthesis and mitochondrial import was disrupted. Cells were mock-infected or infected with WT vaccinia virus in the presence of vehicle (DMSO), TOFA, C75 or etomoxir and pulse labeled with [^35^S]-methionine for 30 min at the indicated times post-infection. As illustrated by the autoradiograph shown in **Fig. 8**, protein synthesis in mock-infected cells was largely unaffected by the inclusion of these inhibitors (lanes 1–4; 4.5 h post-treatment or lanes 9, 11, 12; 7.5 h post-treatment), with the exception of TOFA-treated cells, where protein synthesis was reduced 5-fold at 7.5 h post-treatment (lane 10). In infected cells, we saw the expected reduction in host protein synthesis at both 4.5 and 7.5 hpi (lanes 5–8 and 13–16). Intermediate viral proteins were readily seen at 4.5 hpi (circles in lanes 5–8) under all treatments, although synthesis levels were somewhat decreased in cells treated with TOFA and etomoxir (to 60 and 37% of control levels, respectively). The synthesis of late viral proteins (stars in lanes 13–16) occurred under all treatments, although a reduction in the level of expression was seen in all drug-treated cells. Overall protein synthesis was diminished to 35% of control levels in cells treated with TOFA and 65% in cells treated with either C75 or etomoxir. These data show that the inhibition of palmitate synthesis and mitochondrial import leads to a modest reduction in the rate of protein synthesis, but does not impair host shutoff or the progression through the characteristic phases of vaccinia gene expression [28].

### Viral assembly is inhibited by TOFA and C75

**Figure 9:**
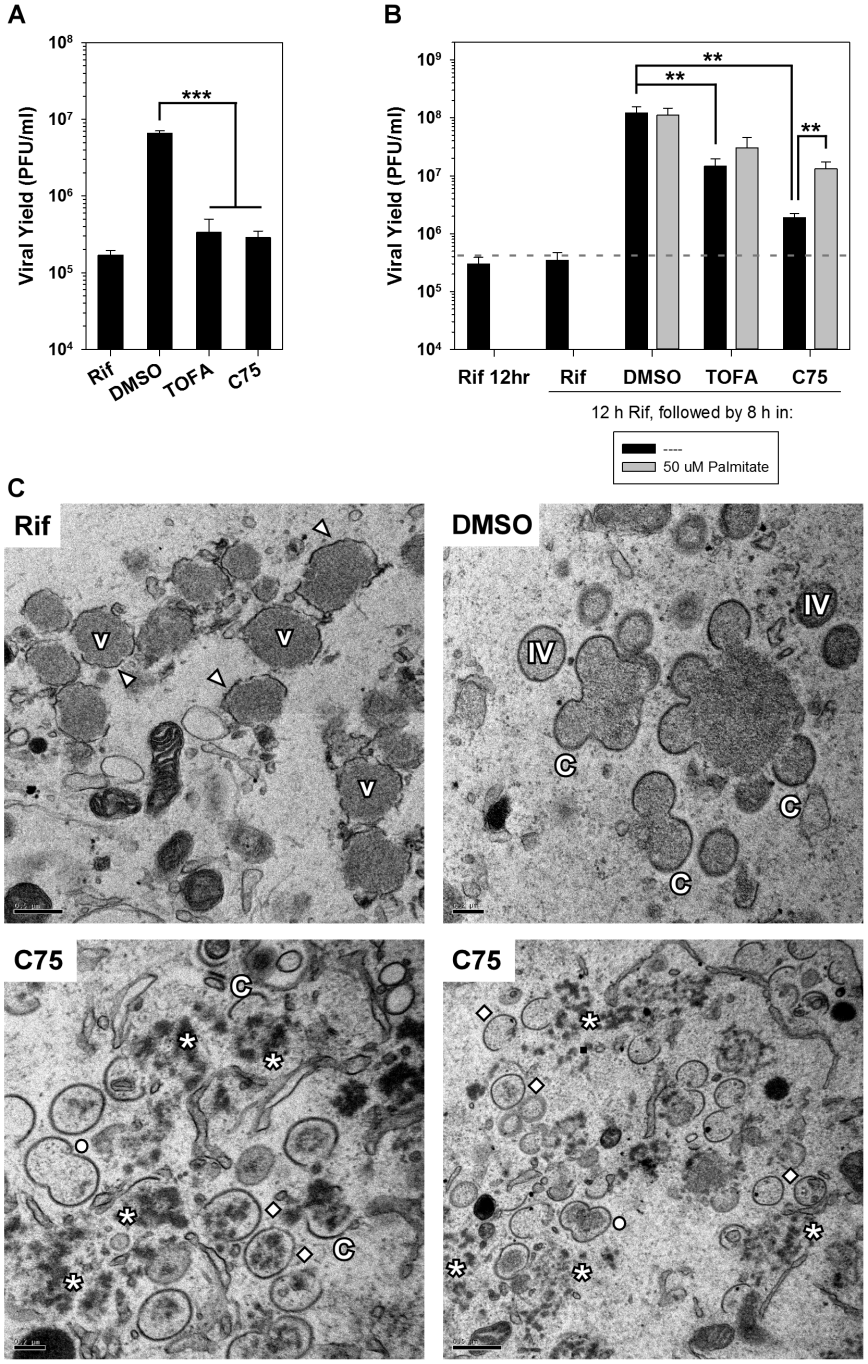
Viral assembly is inhibited significantly by TOFA and C75. A) BSC40 cells were infected with C*ts*28 (MOI 5) for 12 h at the non-permissive temperature (39.7°C) to synchronize the infectious cycle at the initiation of assembly. Cultures were then shifted to the permissive temperature (31.5°C) to relieve the block to assembly and infection was allowed to proceed for an additional 8 h. The shift-down was performed in the presence of rifampicin (rif) (100 µg/ml), a known inhibitor of assembly, DMSO (vehicle control), TOFA (154 µM) or C75 (39 µM). Viral yield is shown; a significant block to virus production is seen in the presence of rif, TOFA or C75 (n = 6) (***, p<0.001). B) BSC40 cells were infected with WT vaccinia virus (MOI 5) in the presence rif (100 µg/ml). At 12 hpi, cells were released from the rif block into media containing rif (100 µg/ml), DMSO, TOFA (154 µM) or C75 (39 µM) in the absence (black bars) or presence (gray bars) of exogenous palmitate (50 µM) for 8 h. Cells were harvested and viral yield analysis reveals that TOFA or C75 exert a significant impact on viral yield that can be partially rescued by the addition of palmitate (n = 6) (**, p<0.02). C) Electron microscopy was utilized to examine specific block in viral assembly seen when rif-arrested cells were released into media containing C75. Cells were treated as in (B) and processed for electron microscopy. Cells maintained in the presence of rif (upper left panel) accumulated large virosomes (V) that were surrounded by flaccid membranes (arrowheads). When cells were released into DMSO (upper right panel), assembly resumed and crescent membranes (C), immature virions (IV) and mature virions (not shown) were abundant. When cells were released into C75 (bottom panels), the virosomal contents dispersed into splotchy aggregates (star); IV, empty IV, and “peanut” shaped double-IV (open circle) were seen.

We next analyzed the process of viral morphogenesis to determine if the inhibition of *de novo* fatty acid biosynthesis affected the formation of mature virions. To focus on morphogenesis itself, without the complications of the minor impact that the inhibitors exert on protein synthesis and genome accumulation, we took two experimental approaches. First, we utilized a temperature-sensitive mutant with a lesion in the F10 protein kinase (C*ts*28); at the non-permissive temperature (39.7°C) the virus has a well-characterized defect in virus assembly [29], [30]. Viral genome replication and the full cycle of gene expression proceed normally, but the infection arrests prior to membrane biogenesis. This arrest is reversed when cells are shifted to permissive conditions (31.5°C) [29]. BSC40 cells were infected with C*ts*28 for 12 h under non-permissive conditions, and then released to permissive conditions to allow the resumption of assembly. Release was performed for 8 h in the presence of vehicle (DMSO), the inhibitor rifampicin (rif), which allows membrane biogenesis to move forward but blocks the formation of immature and mature virions, TOFA, or C75. The viral yield from cells shifted to permissive conditions in the presence of DMSO was 40-fold greater than the yield from cells shifted under conditions in which virus production was inhibited (rif) (**Fig. 9A**). Interestingly, the inclusion of TOFA or C75 after the temperature shift was as deleterious to virus production as rifampicin. These data strongly suggest that *de novo* fatty acid biosynthesis, and by extrapolation ATP production, is particularly important for virion assembly.

To further examine the importance of fatty acid biosynthesis to viral assembly, we performed infections in the presence of rifampicin; the reversible inhibitor rifampicin prevents the interaction of the viral proteins D13 and A17, thereby preventing the assembly of immature virions [31]–[34]. In the presence of rif, DNA replication and the full profile of protein expression proceed normally, but morphogenesis arrests at an early stage [35]. BSC40 cells were infected with WT vaccinia virus in the presence of rif for 12 h, washed with fresh media and incubated for another 8 h in the presence of rif (negative control, to inhibit resumption of assembly), DMSO (positive control, to allow full recovery), TOFA, or C75 (**Fig. 9B**). Cells released from the rif block into DMSO produced 345-fold more virus than those maintained in rif. In cells treated with TOFA or C75, an impaired recovery was seen, with viral production being only 41- and 5-fold greater than observed in cultures maintained in rif (black bars). The addition of palmitate gave a significant but incomplete (7-fold) rescue of virus production in cells released from rif in the presence of C75 (gray bar).

Having shown that C75 (and TOFA) impaired virion assembly even when added after DNA replication and late gene expression had been accomplished, electron microscopic analysis was performed on cells infected in the presence of rif for 12 h and then released into rif, DMSO, or C75 as described above (**Fig. 9B**). The phenotype seen for cells maintained in rif was as expected: flaccid membranes (arrowheads) accumulated around electron-dense virosomes (V) (**Fig. 9C**, upper left). These membranes lack the exterior D13 scaffold and hence do not show the spiked appearance of normal virion membranes, and no progression to typical crescents, immature or mature virions was found. When cells were released into DMSO (upper right panel), the full range of assembly intermediates, including crescents (C), immature virions (IV), and mature virions (not shown) was seen. When cells were released into C75 (lower panels), a novel block in assembly was seen. Crescent membranes, IV membranes, and “peanut” shaped membranes (open circle) resembling two fused IV were seen. However, the smooth, dense viroplasm observed in rif synchronization was dispersed into splotches or aggregates (star), some of which were being enclosed in IV membranes (open diamond). Many of the IVs, however, appeared devoid of interior contents. No mature virions were seen. Consistent with the data shown in **Fig. 9B**, the addition of exogenous palmitate to C75 treated cells partially rescued the block observed in cells treated with C75 alone (data not shown). Taken together, these data show that *de novo* fatty acid biosynthesis is important for virion assembly, even after the constituent components are present. The most dramatic impairment observed was the fragmentation of the virosomal contents into splotchy aggregates, and the inefficient incorporation of this material into nascent virion membranes.

## Discussion

**Figure 10:**
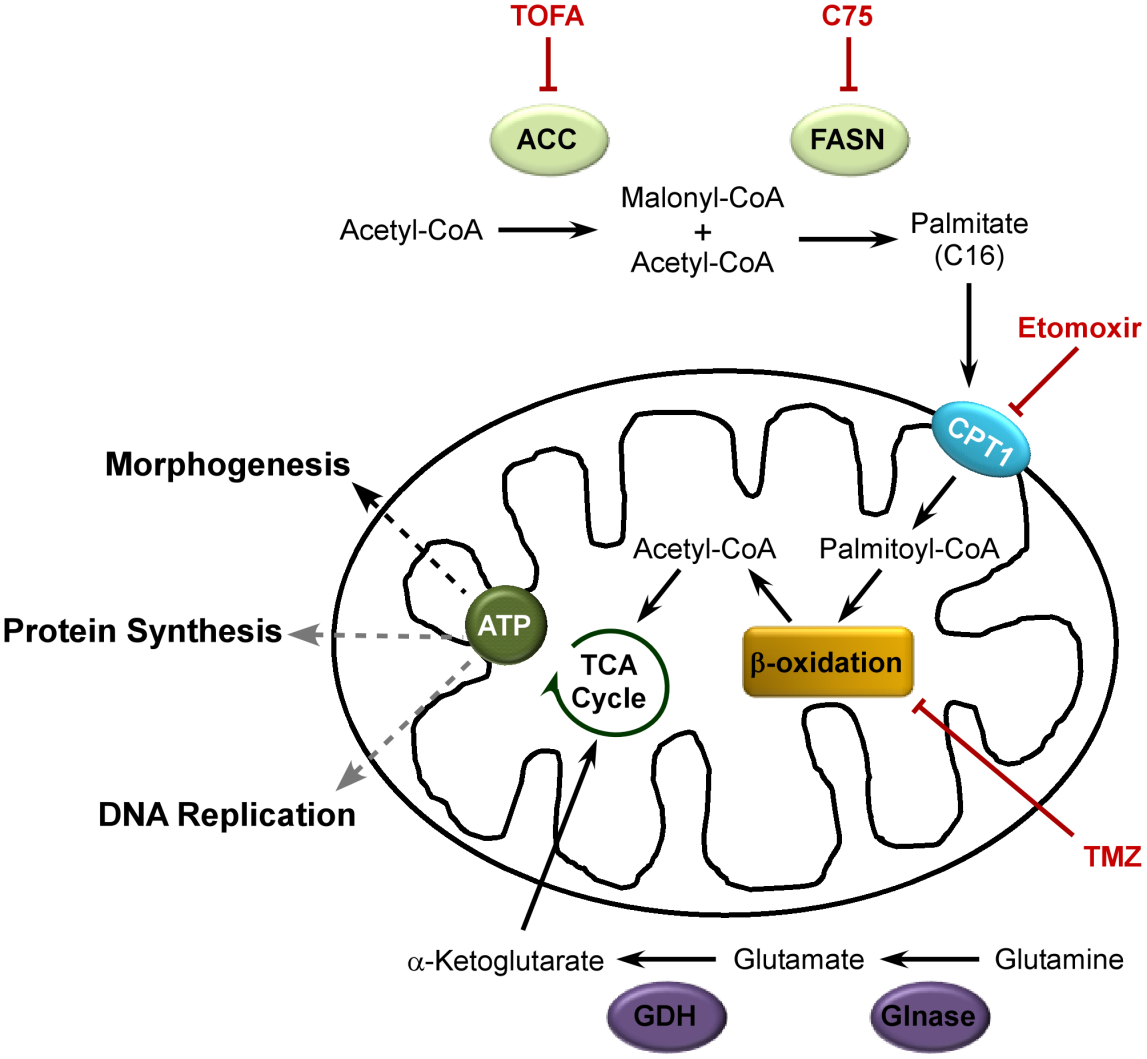
Model for palmitate utilization during vaccinia virus infection. The *de novo* fatty acid biosynthetic pathway is utilized to generate ATP during vaccinia virus infection. ACC (acetyl-CoA carboxylase) converts acetyl-CoA to malonyl-CoA; FASN (fatty acid synthease) catalyzes successive condensation reactions of malonyl CoA with acetyl-CoA to generate the 16-carbon fatty acid, palmitate. Palmitate is then imported into the mitochondria by CPT1 (carnitine palmitoyl transferase) 1. Within the mitochondria, palmitate undergoes β-oxidation to generate acetyl-CoA which drives the TCA cycle (and subsequent oxidative phosphorylation) to generate ATP. Glutaminolysis enables glutamine to anaplerotically fill in the TCA cycle. Glutamine is converted to glutamate by Glnase (glutaminase) which is further converted to α-ketoglutarate by GDH (glutamate dehydrogenase). Inhibitors of this pathway are shown in red; impairment of each of the steps in this pathway significantly diminishes vaccinia virus production. ATP production supports DNA replication and protein synthesis (gray dashed lines), but is most important for the assembly of nascent virions (black dashed line).

The data presented here support a model that is depicted schematically in Figure 10. The *de novo* fatty acid biosynthetic pathway, comprising the two enzymes ACC and FASN, generates palmitate within the cytoplasm. Palmitate is then imported into the mitochondria by CPT1 where it undergoes β-oxidation to generate acetyl-CoA. The generation of acetyl-CoA from palmitate, rather than glucose, drives the TCA cycle to generate sufficient amounts of ATP for maximal viral production. Inhibition of any part in this pathway causes a significant diminution in viral yield. ATP generated by this pathway contributes to viral DNA replication and protein synthesis, but is most important for the morphogenesis of infectious virions. Glucose is largely dispensable for infection, but glutamine is essential. A major role for glutamine is to enable the anaplerotic support of the TCA cycle via deamination to glutamate within the cytoplasm, and conversion to α-ketoglutarate within the mitochondria, by the successive activities of glutaminase and glutamate dehydrogenase. The robust action of the TCA cycle may allow for the shuttling of citrate into the cytoplasm to generate pools of acetyl-CoA for the synthesis of palmitate.

Having previously shown that the FASN inhibitor cerulenin impaired vaccinia virus morphogenesis [13], we began the current study with the expectation that *de novo* fatty acid biosynthesis would be needed to augment the intracellular membranes available to support viral assembly. However, this expectation was not supported by further experimentation. For example, triacsin C, which inhibits long chain acyl-CoA synthetase and drastically impairs phospholipid synthesis, has no impact on vaccinia virus infection (**Fig. 2B**). Because cerulenin, TOFA and C75 all inhibited vaccinia virus infection, we considered what other roles the end-product of their activity, palmitate, might play. We were able to discard an essential role for protein palmitoylation in the progression of the life cycle by demonstrating that infection was resistant to 2-bromopalmitate (**Fig. 2A**). There are several viral proteins that do undergo palmitoylation, including A33, B5 and F13 [36], [37], but these proteins have been shown to participate in the maturation of a small subset of mature virions (MV) into extracellular virions (EV); our studies are focused on the production of MV, which constitute the vast majority of virions and are sufficient for infection in tissue culture.

The third key role for palmitate is to undergo β-oxidation within mitochondria and generate acetyl-CoA, a key component of the TCA cycle, which in turn drives oxidative phosphorylation and ATP generation. It seems intuitive that vaccinia infection would require high levels of ATP, given the robust synthesis of viral RNA, DNA and protein and the assembly of large numbers of complex virions. We found that glucose was dispensable for infection (**Fig. 3A**). Moreover, we also found that oxygen consumption, a surrogate measure of mitochondrial ATP synthesis, was elevated significantly in infected cells (**Fig. 5A**). This observation was consistent with a previous report demonstrating an increase in ATP levels in vaccinia-infected HeLa cells [38]. Our data indicate that optimal infection requires the synthesis, mitochondrial import and β-oxidation of palmitate (**Figs. 1** **and** **3**). It should be noted that complete oxidation of one molecule of palmitate yields more ATP than can be generated from one molecule of glucose (glucose→2 acetyl-CoA+36 ATP; palmitate→8 acetyl-CoA+129 ATP). Even considering the fact that generating palmitate requires 57 ATP (15 ATP to generate and activate acetyl-CoA and 42 ATP to complete 7 condensation reactions), palmitate yields twice the amount of ATP compared to glucose. Interestingly, this value correlates with the ∼2-fold increase in OCR we observe upon infection with vaccinia virus. Thus, at least in BSC40 cells, vaccinia virus is more dependent upon β-oxidation of fatty acids within the mitochondria than on glycolysis for maximal ATP production. This altered metabolic profile is an early event during infection, since OCR levels were already high by 1.5 hpi (**Fig. 5**). Determining how mitochondrial activity is stimulated early in infection is an area of interest for future study.

Given the central role of FASN in the generation of palmitate, we considered how vaccinia might exploit or maximize its activity. HCV has been shown to upregulate expression of the enzymes involved in fatty acid synthesis [7]–[10]; however, the levels of FASN are not upregulated during vaccinia infection (data not shown). It will be of interest in the future to determine whether the specific activity of ACC or FASN is upregulated upon vaccinia infection, since both enzymes are known to be modulated by phosphorylation, ubiquitination and by specific interactions with stimulatory and inhibitory binding partners [39]–[46]. DV and HCV induce a change in the localization of FASN to the sites of RNA replication [6], [11], but the localization of FASN does not appear to change during vaccinia infection, and we have not been successful in detecting any stable and/or obvious interactions between FASN and vaccinia-encoded proteins (not shown). However, we were surprised to see that, in BSC40 cells, a significant portion of FASN co-localizes with mitochondria (**Fig. 6**); this co-localization has not been reported before. It is reasonable to conclude that this proximity would facilitate the mitochondrial import of palmitate immediately after synthesis. Electron microscopic analysis of vaccinia-infected cells has revealed that mitochondria are numerous at the periphery of viral factories, which may facilitate the delivery of ATP to viral machinery.

The *de novo* fatty acid biosynthetic pathway driven by ACC and FASN requires ample concentrations of acetyl-CoA, which can be generated via two primary pathways. The first mechanism involves the catabolism of pyruvate by pyruvate dehydrogenase. This is unlikely, however, since it has recently been reported that a ‘pseudo-hypoxic’ state is induced early after vaccinia infection [12], which leads to an up-regulation of HIF1α-dependent genes causing an increase in the flux of glucose to lactate and concomitant decrease in accumulation of acetyl-CoA in the mitochondria [47]. The second mechanism involves the catabolism of citrate to acetyl-CoA and oxaloacetate by ATP citrate lyase (ACLY). This pathway is utilized by highly proliferative cells that utilize citrate as an intermediate for lipogenic pathways. In order for the TCA cycle not to slow down as a result of this diversion of citrate for fatty acid synthesis, replenishment must occur. In HCMV-infected cells, replenishment of the TCA cycle has been shown to occur through glutaminolysis and anaplerosis [48]. Glutaminolysis refers to the use of glutamine as a source for the ultimate synthesis of α-ketoglutarate, which can subsequently be utilized in the synthesis of citrate. Indeed, glutamine is essential for vaccinia virus infection: depletion of glutamine reduces viral yield by ∼1300-fold (**Fig. 4A**). Oxaloacetate and α-ketoglutarate fill the TCA cycle and are able to rescue viral yield significantly, albeit only in the presence of a low concentration of glutamine (**Fig. 4B**). This suggests that citrate may indeed provide the acetyl-CoA that is needed in the cytoplasm of vaccinia-infected cells for palmitate synthesis. Further metabolomic studies aimed at determining changes in the levels of acetyl-CoA, malonyl-CoA, palmitate and TCA cycle intermediates during vaccinia infection will be highly informative. It is perhaps not surprising that productive viral infection requires a low level of glutamine that cannot be rescued by TCA cycle intermediates, because glutamine is known to play roles in purine and pyrimidine synthesis as well as protein synthesis.

Our studies indicated that the progression of the viral life cycle through three temporally regulated stages of gene expression, and through the process of viral DNA synthesis and maturation, was not impaired in the presence of C75, TOFA, or etomoxir. We observed a mild decrease in the levels of genomes that accumulated (**Fig. 7**), and a modest decrease in the levels of proteins being synthesized (**Fig. 8**). Even when C75 or TOFA were added at 12 hpi, when these biosynthetic processes are largely complete, they had a significant impact on virus production. The process of virion morphogenesis appears to be acutely sensitive to the impact of these drugs and the attendant decrease in ATP synthesis, as demonstrated by our use of C*ts*F10 and rifampicin to arrest cells at the onset of morphogenesis and then monitor virus production when these blocks were released (**Fig. 9A and B**). When cells were released into C75 or TOFA, the expected burst of virus production was diminished by >10-fold.

We utilized electron microscopy to visualize the assembly process after synchronization with rifampicin for 12 h and release into vehicle alone or C75 for an additional 8 h (**Fig. 9C**). When cells were released into vehicle alone, we saw the full spectrum of assembly intermediates including crescents (C), immature virions (IV), immature virions with nucleoids (IVN) and mature virions (MV). The images seen upon release into C75 were strikingly different. The flaccid membranes that accumulate in the presence of rifampicin were indeed “chased” into normal crescent membranes by their association with the D13 scaffold protein. Many of these crescents were enlarged and resembled IV, although a significant number had a “peanut” shape reminiscent of two IVs that had fused together. However, most of these IVs and crescents were “empty” and not associated with smooth virosomal material. The large virosomes that accumulate in the presence of rifampicin were dispersed into fragmented aggregates, as if the solubility of these virosomal proteins was compromised. This phenotype was quite distinct from what has been observed before, and suggests that the solubility of virosomal proteins and their inclusion in nascent virions is highly dependent upon sufficient ATP levels.

In sum, our data support the conclusion that the *de novo* fatty acid biosynthetic pathway plays a key role in viral infection by generating sufficient palmitate for import into mitochondria. The subsequent ß-oxidation of palmitate can drive the TCA cycle and augment the production of ATP; data generated using Seahorse technology confirms that ATP production is highly elevated within infected cells in a manner that depends upon mitochondrial fatty acid import. Thus, the vaccinia infectious cycle is highly dependent upon cellular bioenergetics; moreover, infection seems to shift cellular metabolism towards a more oxidative and less glycolytic state. The *de novo* fatty acid biosynthesis pathway offers a novel target for developing therapeutics to treat poxvirus infections. Interestingly, this biochemical pathway is often upregulated in cancer [49], and inhibitors that target this pathway (such as TOFA, C75, and cerulenin) inhibit cancer cell proliferation *in vitro*. New compounds are being developed that retain efficacy but show increased solubility and reduced toxicity, such as the FASN inhibitor, G28UCM [50]–[52]. It will be interesting to determine if such compounds would have anti-poxviral efficacy in tissue culture and animal models.

## Materials and Methods

### Reagents

[^35^S]-methionine and [^3^H]-palmitate were purchased from Perkin Elmer Life Sciences (Boston, MA). [^14^C]-labeled protein molecular weight markers and Mitotracker Red CMXRos were purchased from Invitrogen (Carlsbad, CA). Protran nitrocellulose membranes were obtained from GE Healthcare Life Sciences (Buckinghamshire, UK). 5-(Tetradecyloxy)-2-furoic acid (TOFA) and triacsin C were obtained from Enzo Life Sciences. C75, 2-bromopalmitate, trimetazidine, rifampicin, fatty-acid free bovine serum albumin, sodium palmitate, oleic acid, glucose, glutamine, oxaloacetate and dimethyl-α-ketoglutaric acid were obtained from Sigma (Saint Louis, MO). Etomoxir was obtained from Tocris Bioscience (Bristol, UK).

### Cell culture and vaccinia virus infections

Monolayer cultures of African green monkey BSC40 cells were maintained at 37°C in Dulbecco modified eagle medium (DMEM; Invitrogen) containing 5% fetal calf serum (FCS) unless otherwise specified. Confluent 35 mm dishes of BSC40 cells were infected with WT vaccinia virus (MOI of 5) in the presence of various pharmacological inhibitors [TOFA (154 µM) and C75 (39 µM), various concentrations of 2-bromopalmitate, various concentrations of triacsin C, etomoxir (360 µM), or various concentrations of trimetazidine] as well as exogenous palmitic acid (50 µM) as indicated, incubated at 37°C for 16 h, harvested and analyzed for viral yield and protein expression. Cell viability was monitored visually following all treatments. At the doses reported herein, no significant cell death was observed as determined by cell morphology and adherence. Viral yield was determined by performing plaque assays on BSC40 cells. Viral yield was plotted as the average of six experiments with error bars representing standard error of the mean. A student two-tailed *t-test* was employed to determine significance.

#### A) Specific morphogenesis blockade and release

(i) BSC40 cells were infected with C*ts*28 [29] (MOI of 5), and incubated at non-permissive temperature (39.7°C) for 12 h. Cells were then shifted to the permissive temperature (31.5°C) for 8 h in the presence of TOFA (154 µM), C75 (39 µM) or rif (100 µg/ml) or vehicle alone (DMSO) followed by analysis of viral yield and protein accumulation. (ii) Alternatively, cells were infected with WT virus (MOI of 5) and incubated at 37°C for 12 h in the presence of rif (100 µg/ml). Rifampicin was then washed out and cells were treated with fresh media containing rif (100 µg/ml), TOFA (154 µM) C75 (39 µM) or vehicle alone (DMSO), as well exogenous palmitic acid as indicated, incubated for 8 h at 37°C, harvested and analyzed for viral yield and protein expression.

#### B) Inhibition of metabolic pathways

(i) BSC40 cells were seeded and infected as above. Cells were washed with glucose-free media (Gibco) and then incubated for 16 h at 37°C in glucose-free media or glucose-free media supplemented with 4 mM glucose. Cells were harvested and analyzed for viral yield. (ii) BSC40 cells were seeded and infected as above. Cells were washed with glutamine-free media (Gibco) and then incubated for 16 h at 37°C in glutamine-free media, glutamine-free media supplemented with various concentrations of glutamine, 15 mM oxaloacetate or 5 mM dimethyl-α-ketoglutaric acid. Cells were harvested and analyzed for viral yield.

### Determination of oxygen consumption rate (OCR)

BSC40 cells were seeded at 4×10^4^ cells per well in a 96-well V-3 PET tissue culture plate (Seahorse Bioscience) and incubated for 24 h at 37°C. Cells were then either mock infected or infected with WT vaccinia virus (MOI of 5). Additionally, cells infected with WT vaccinia virus were treated with etomoxir (360 µM) or vehicle control for the duration of the experiment. Prior to the experiment, the cells were incubated for 30 min in High Glucose DMEM (Gibco) lacking sodium bicarbonate and supplemented with 10 mM HEPES (Gibco) and drugs as appropriate. Oxygen consumption rates were measured every 8.2 minutes using the Seahorse Bioscience XF-96 extracellular flux analyzer (Seahorse Bioscience) from 1.5 to 12 hpi at 37°C. Data were assayed in quadruplicate on three separate days and graphed in Microsoft Excel. Error bars represent standard error of the mean. Linear regression analysis was employed to show a statistical difference between sample groups.

### Immunofluorescence microscopy

Confluent 4-well chamber slides of BSC40 cells were either mock infected or infected with WT vaccinia virus (MOI of 2) for 7 h at 37°C and analyzed for FASN localization and replication foci as described below.

#### A) Determination of FASN localization

At 6 hpi, cells were treated with Mitotracker Red (100 nM) for 1 h at 37°C. Cells were washed twice with cold PBS on ice, fixed in cold methanol∶acetone (1∶1) for 15 min on ice and washed twice with cold PBS. Cells were permeabilized in Saponin Buffer (1% BSA; 0.3 M Glycine; 0.1% saponin in PBS) for 1 h at room temperature. Samples were then stained with polyclonal anti-FASN (AbCam 22759) antiserum diluted in Saponin Buffer followed by a secondary antibody conjugated to AlexaFlour488 (goat anti-rabbit IgG) (Invitrogen) diluted in Saponin Buffer. DAPI was added for 15 min at room temperature. As indicated, a FASN blocking peptide (1 mg/ml) (AbCam 25719) was incubated in Saponin Buffer with the polyclonal anti-FASN antiserum. Samples were mounted with Vectashield (Vector Laboratories, Inc. Burlingam, CA) and pictures were captured using a Nikon Eclipse TE2000-U microscope and NIS-Elements BR3.2 software (Tokyo, Japan). Pearson's coeeficient was utilized as a measure of the degree of colocalization between FASN and Mitotracker.

#### B) Analysis of replication foci

Virally infected cells were maintained in the presence of bromodeoxyuridine (BrdU) (25 µg/ml) as well as vehicle (DMSO), TOFA (154 µM) or etomoxir (360 µM). Cells were washed twice with cold PBS on ice, fixed in cold 4% paraformaldehyde in PBS for 15 min on ice and washed twice with cold PBS. Cells were then permeabilized with 0.2% TritonX-100 in PBS for 5 min on ice. Samples were then stained with monoclonal anti-BrdU (5 µg/ml; Developmental Studies Hybridoma Bank, University of Iowa, Iowa City, IA) and polyclonal anti-I3 (1∶400) [53] antibodies followed by secondary antibodies conjugated to either AlexaFluor 594 (goat anti-mouse IgG) or AlexaFluor 488 (goat anti-rabbit IgG) (Invitrogen). DAPI was added for 15 min at room temperature. Cells were mounted and pictures were captured as described above.

#### C) Accumulation of lipid droplets

Confluent 4-well chamber slides of BSC40 cells were treated with 400 µM oleic acid, to induce lipid droplet formation, in the absence or presence of triacsin C (6.25 µM) for 20 h. Cells were washed twice with cold PBS, fixed with cold 4% paraformaldehyde in PBS for 15 min and washed twice with cold PBS. Cells were permeabilized with 0.2% TritonX-100 in PBS for 5 min on ice and then stained with BODIPY493/503 (Life Technologies, NY) for 1 h at room temperature. DAPI was added for 15 min at room temperature. Slides were mounted and pictures were captured as described above.

### [^3^H]-palmitate labeling to assess palmitoylation

Confluent monolayers of BSC40 cells were pretreated with DMSO or 50 µM 2-bromopalmitate for 1.5 h. Cells were washed twice with 1 ml wash medium (DMEM; 5% dialyzed FBS; 3.6 mg/ml fatty acid-free BSA; 5 mM sodium pyruvate) and then incubated with the same medium supplemented with 0.5 mCi/ml [^3^H]-palmitate for 4 h at 37°C. Cells were harvested on ice, washed with cold PBS and lysed in RIPA buffer (20 mM Tris, pH 7.5; 137 mM NaCl; 2 mM EDTA, 0.5% sodium deoxycholate; 0.1% SDS; 1% TritonX-100; 10% glycerol and protease inhibitors) for 20 min on ice. Whole cell lysates were resolved by SDS-PAGE and visualized by fluorography.

### Pulsed-field gel electrophoresis (PFGE)

Confluent 35 mm dishes of BSC40 cells were infected with WT vaccinia virus (MOI of 5) for 10 h in the presence of vehicle control, TOFA (154 µM), C75 (39 µM) or etomoxir (360 µM). Cell pellets were processed as described earlier [54]. Briefly, cell pellets were embedded in 0.5% low-melting-point agarose and digested for 48 h at 50°C with mild agitation in 500 µl of ESP buffer (1% sarcosyl, 0.5 M EDTA [pH 9.0], 0.5 mg/ml proteinase K). The plugs were equilibrated in 0.5× TBE buffer prior to insertion into premolded wells of a 1% SeaKem Gold agarose gel cast and resolved in the same buffer. The DNA was resolved on a CHEF Mapper XA apparatus (Bio-Rad) at 6 V/cm for 5 hours at 14°C, using a switching time gradient of 0.05 to 17 seconds, a 2.84 ramping factor (non-linear), and a 120° angle. The DNA was visualized by ethidium bromide staining and the image was captured and quantified using AlphaView software (ProteinSimple).

### [^35^S]-methionine labeling to assess viral protein synthesis

Confluent 35 mm dishes of BSC40 cells were infected with WT virus (MOI of 5) or mock infected and treated with TOFA (154 µM), C75 (39 µM), etomoxir (360 µM) or vehicle control. Cells were then rinsed with methionine-free DMEM and pulsed with 100 µCi/ml [^35^S]-methionine for 30 min at either 4 hpi or 7 hpi. Cells were harvested, rinsed with PBS containing protease inhibitors, resuspended in 250 µl PBS containing 1× PSB and protease inhibitors and resolved on a SDS-10–17% acrylamide gel. The gel was then stained with coomassie brilliant blue and subjected to autoradiography. Effects on protein synthesis were quantified by measuring the radioactivity present in each lane using Typhoon FLA 7000 imager and Image Quant TL imaging software (GE Healthcare Bio-Sciences, Pittsburgh, PA). The signals in the experimental lanes were normalized to the DMSO control sample for each time point.

### Electron microscopy

Confluent 60 mm dishes of BSC40 cells were infected with WT virus in the presence of rifampicin and released as described above. Eight hours post release, samples were fixed *in situ* with 1% glutaraldehyde in 0.1 M Sorensen's phosphate buffer (pH 7.4) and processed for epoxy embedding and conventional transmission electron microscopy. Sections were examined on a JEOL JEM 2100 electron microscope and digital images were acquired using a GATAN Ultrascan 1000 camera.

### Preparation of digital figures

Original data were scanned on an Epson Perfection scanner (Long Beach, CA) and were adjusted with Adobe Photoshop software (Adobe Systems, Inc., San Jose, CA). Statistical analysis and graph preparation were performed using SigmaPlot software (Systat Software, Chicago, IL) or GraphPad Prism (La Jolla, CA). Final figures were assembled and labeled with Canvas software (Deneba Systems, Miami, FL).

## Supporting Information

Figure S1 **Confirmation that 2-bromopalmitate and triacsin C inhibit palmitoylation and fatty acid acylation, respectively.** A) Confluent monolayers of BSC40 cells were treated with DMSO or 50 µM 2-bromopalmitate (2-BP) in the presence of [^3^H]-palmitate for 4 h. Whole cell lysates were resolved and exposed to fluorography. B) BSC40 cells were treated for 20 h with DMSO or 6.25 µM triacsin C in the presence of 400 µM oleic acid to induce the formation of lipid droplets. Cells were fixed with 4% PFA and stained with BODIPY493/503 to mark lipid droplets as well as DAPI to mark nuclei.
(TIF)
