# Supplementary figures and images for: De novo Fatty Acid Biosynthesis Contributes Significantly to Establishment of a Bioenergetically Favorable Environment for Vaccinia Virus Infection
Source: PLoS Pathog. 2014 Mar 20;10(3):e1004021. doi: 10.1371/journal.ppat.1004021 (PMC3961357; doi:10.1371/journal.ppat.1004021)

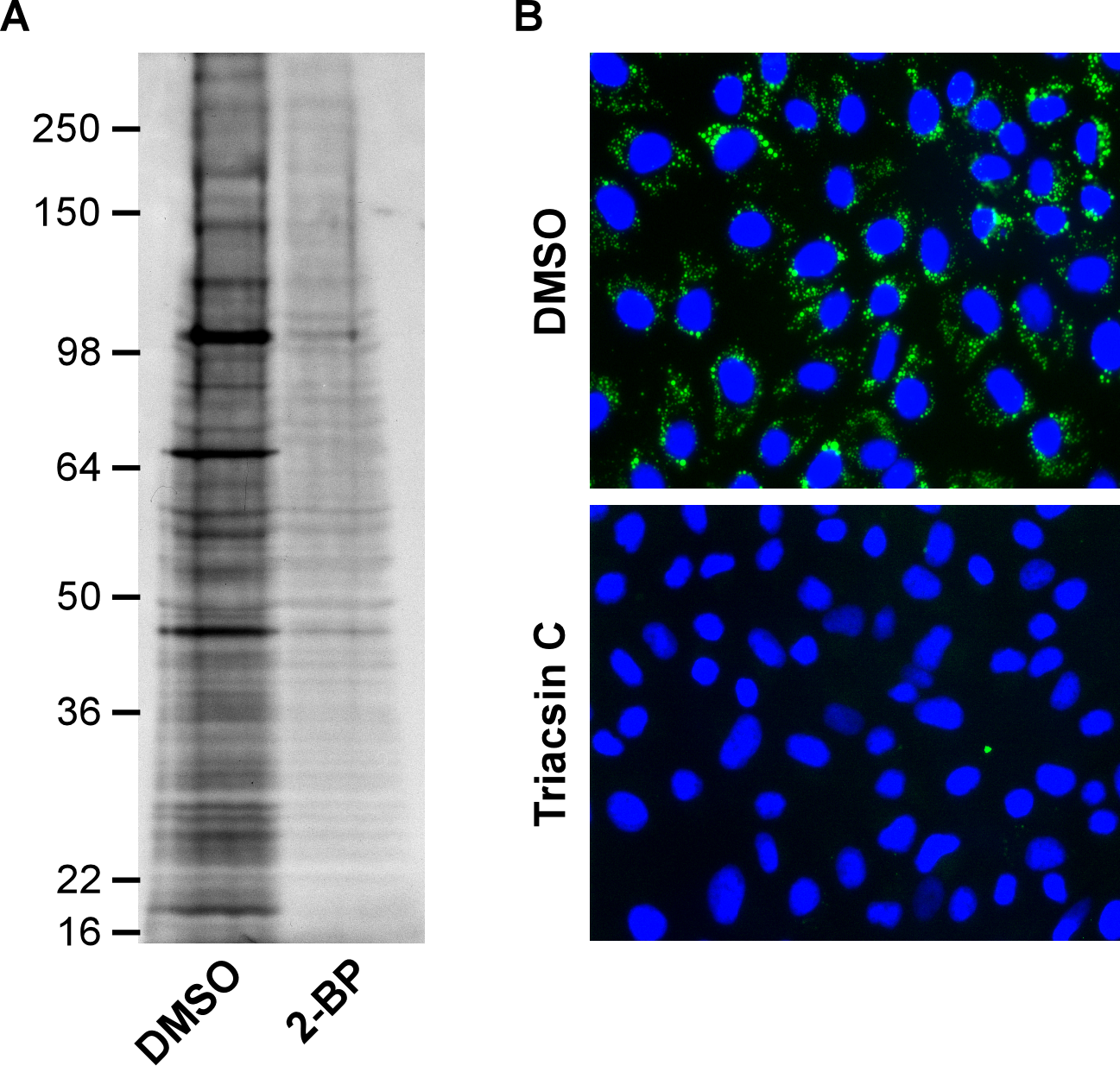

Supplement: Figure S1 — Confirmation that 2-bromopalmitate and triacsin C inhibit palmitoylation and fatty acid acylation, respectively. A) Confluent monolayers of BSC40 cells were treated with DMSO or 50 µM 2-bromopalmitate (2-BP) in the presence of [3H]-palmitate for 4 h. Whole cell lysates were resolved and exposed to fluorography. B) BSC40 cells were treated for 20 h with DMSO or 6.25 µM triacsin C in the presence of 400 µM oleic acid to induce the formation of lipid droplets. Cells were fixed with 4% PFA and stained with BODIPY493/503 to mark lipid droplets as well as DAPI to mark nuclei. (TIF) [file ppat.1004021.s001.tif]
